# (*E*)-2-[(1*H*-Imidazol-4-yl)methyl­idene]hydrazinecarbo­thio­amide monohydrate

**DOI:** 10.1107/S1600536813022927

**Published:** 2013-08-23

**Authors:** Benayad Houari, Samira Louhibi, Leila Boukli-Hacene, Thierry Roisnel, Mustapha Taleb

**Affiliations:** a Laboratoire de Chimie Inorganique et Environnement, Université de Tlemcen, BP 119, 13000, Tlemcen, Algeria; b Centre de Diffractométrie X, UMR 6226 CNRS, Unité Sciences Chimiques de Rennes, Université de Rennes I, 263 Avenue du Général Leclerc, 35042 Rennes, France; c USMBA, FSDM, Département de Chimie, BP 1796 Fès - Atlas, Morocco

## Abstract

In the title compound, C_5_H_7_N_5_S·H_2_O, the main mol­ecule is approximately planar, with a maximum deviation from the mean plane through the non-H atoms of 0.1478 (12) Å for the amine N atom. In the crystal, the components are connected *via* N—H⋯O, N—H⋯S and O—H⋯N hydrogen bonds, forming a three-dimensional network.

## Related literature

For the biological activity of thio­simecarbazone derivatives, see: Finch *et al.* (2000 ▶). For the crystal structures of related compounds, see: Alomar *et al.* (2013 ▶).
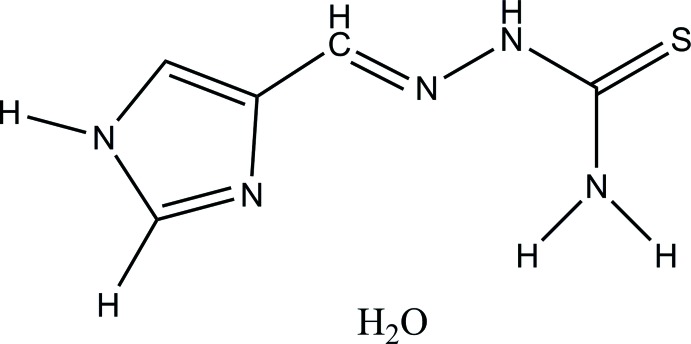


## Experimental

### 

#### Crystal data

C_5_H_7_N_5_S·H_2_O
*M* *_r_* = 187.23
Monoclinic, 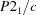

*a* = 10.8734 (5) Å
*b* = 11.2416 (5) Å
*c* = 7.0822 (3) Å
β = 75.601 (2)°
*V* = 838.50 (6) Å^3^
*Z* = 4
Mo *K*α radiation
μ = 0.35 mm^−1^
*T* = 150 K
0.4 × 0.23 × 0.16 mm

#### Data collection

Bruker APEXII CCD diffractometer
6537 measured reflections
1903 independent reflections
1726 reflections with *I* > 2σ(*I*)
*R*_int_ = 0.031

#### Refinement

*R*[*F* ^2^ > 2σ(*F* ^2^)] = 0.030
*wR*(*F* ^2^) = 0.083
*S* = 1.06
1903 reflections
127 parameters
H-atom parameters not refined
Δρ_max_ = 0.38 e Å^−3^
Δρ_min_ = −0.22 e Å^−3^

### 

Data collection: *APEX2* (Bruker, 2006 ▶); cell refinement: *SAINT* (Bruker, 2006 ▶); data reduction: *SAINT*; program(s) used to solve structure: *SIR97* (Altomare *et al.*, 1999 ▶); program(s) used to refine structure: *SHELXL97* (Sheldrick, 2008 ▶); molecular graphics: *ORTEP-3 for Windows* (Farrugia, 1999 ▶); software used to prepare material for publication: *WinGX* publication routines (Farrugia, 2012 ▶) and *CRYSCAL* (T. Roisnel, local program).

## Supplementary Material

Crystal structure: contains datablock(s) I, publication_text. DOI: 10.1107/S1600536813022927/lh5642sup1.cif

Structure factors: contains datablock(s) I. DOI: 10.1107/S1600536813022927/lh5642Isup2.hkl

Supplementary material file. DOI: 10.1107/S1600536813022927/lh5642Isup3.docx

Supplementary material file. DOI: 10.1107/S1600536813022927/lh5642Isup4.cml

Additional supplementary materials:  crystallographic information; 3D view; checkCIF report

## Figures and Tables

**Table 1:** Hydrogen-bond geometry (Å, °)

*D*—H⋯*A*	*D*—H	H⋯*A*	*D*⋯*A*	*D*—H⋯*A*
N1—H1*A*⋯O21	0.92 (2)	2.34 (2)	3.2325 (16)	163.2 (18)
N8—H8⋯S1^i^	0.90 (2)	2.51 (2)	3.3334 (13)	153.0 (18)
O21—H21*B*⋯N10	0.87 (2)	2.17 (2)	3.0399 (15)	172 (2)

Symmetry code: (i) 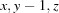.

**Fig. 1.**
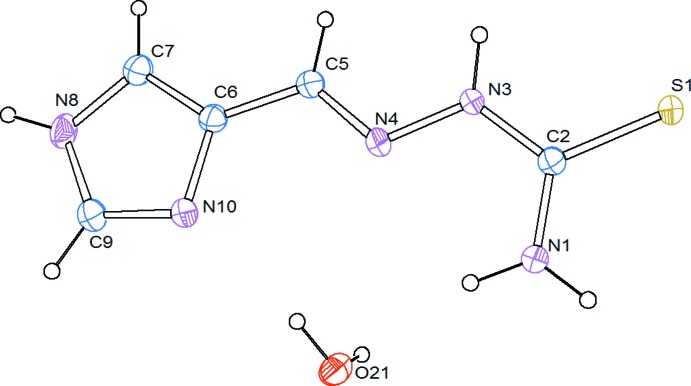
The molecular structure of the title compound. Displacement ellipsoids are drawn at the 50% probability level.

**Fig. 2.**
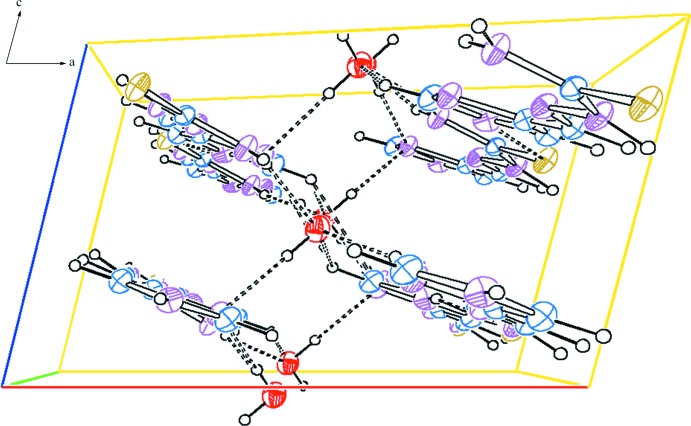
Part of the crystal structure with hydrogen bonds shown as dashed lines.

## supplementary crystallographic information

### 1. Comment

Our interest in thiosemicarbazone derivatives stems from their wide spectrum of biological activity (Finch *et al.*, 2000; Alomar *et al.* 2013). As part of our study of thiosemicarbazone derivatives, we report herein the crystal structure of the title compound (I). The molecular structure of (I) is shown in Fig. 1. The molecule is approximately planar and the maximum deviation from the least squares plane through the 11 non-hydrogen atoms is -0.1478 (12) Å for N1. The bond angles suggest *sp*^2^ hybridization for the C and N atoms which contributes to the planarity of the molecule. The crystal packing is stabilized by intermolecular N—H···O and N—H···S hydrogen bonds (Fig. 2 and Table 1) forming a three-dimensional network.

### 2. Experimental

All the chemicals were purchased from Merck and were used as received. An equimolar amount of thiosemicarbazide 10 mmol (0.91 g) and imidazolecarboxaldehyde 10 mmol (0.96 g) were dissolved in a mixture of ethanol and water (30 ml, 50%) and refluxed for 5 h in the presence of a catalytic amount of glacial acetic acid. Yellow crystals suitable for X-ray analysis were obtained after slow evaporation of the solution.

### 3. Refinement

H atoms bonded to C atoms were placed in calculated positions with C—H = 0.95 Å and refined in a riding-model approximation with *U*_iso_(H) = 1.2*U*_eq_(C). The H atoms bonded to O and N atoms were refined independently with fixed isotropic displacement parameters.

### Crystal data

**Table:**

C_5_H_7_N_5_S·H_2_O	*D*_x_ = 1.483 Mg m^−^^3^
*M**_r_* = 187.23	Melting point: 0 K
Monoclinic, *P*2_1_/*c*	Mo *K*α radiation, λ = 0.71073 Å
*a* = 10.8734 (5) Å	Cell parameters from 3274 reflections
*b* = 11.2416 (5) Å	θ = 2.7–27.5°
*c* = 7.0822 (3) Å	µ = 0.35 mm^−^^1^
β = 75.601 (2)°	*T* = 150 K
*V* = 838.50 (6) Å^3^	Prism, colourless
*Z* = 4	0.4 × 0.23 × 0.16 mm
*F*(000) = 392	

### Data collection

**Table:**

Bruker APEXII CCD diffractometer	*R*_int_ = 0.031
Graphite monochromator	θ_max_ = 27.5°, θ_min_ = 3.5°
CCD rotation images, thin slices scans	*h* = −13→14
6537 measured reflections	*k* = −14→13
1903 independent reflections	*l* = −9→9
1726 reflections with *I* > 2σ(*I*)	

### Refinement

**Table:**

Refinement on *F*^2^	Primary atom site location: structure-invariant direct methods
Least-squares matrix: full	Secondary atom site location: difference Fourier map
*R*[*F*^2^ > 2σ(*F*^2^)] = 0.030	Hydrogen site location: inferred from neighbouring sites
*wR*(*F*^2^) = 0.083	H-atom parameters not refined
*S* = 1.06	*w* = 1/[σ^2^(*F*_o_^2^) + (0.0438*P*)^2^ + 0.2645*P*] where *P* = (*F*_o_^2^ + 2*F*_c_^2^)/3
1903 reflections	(Δ/σ)_max_ = 0.003
127 parameters	Δρ_max_ = 0.38 e Å^−^^3^
0 restraints	Δρ_min_ = −0.22 e Å^−^^3^

### Special details

**Table:**

Geometry. All esds (except the esd in the dihedral angle between two l.s. planes) are estimated using the full covariance matrix. The cell esds are taken into account individually in the estimation of esds in distances, angles and torsion angles; correlations between esds in cell parameters are only used when they are defined by crystal symmetry. An approximate (isotropic) treatment of cell esds is used for estimating esds involving l.s. planes.
Refinement. Refinement of F^2^ against ALL reflections. The weighted R-factor wR and goodness of fit S are based on F^2^, conventional R-factors R are based on F, with F set to zero for negative F^2^. The threshold expression of F^2^ > 2sigma(F^2^) is used only for calculating R-factors(gt) etc. and is not relevant to the choice of reflections for refinement. R-factors based on F^2^ are statistically about twice as large as those based on F, and R- factors based on ALL data will be even larger.

### Fractional atomic coordinates and isotropic or equivalent isotropic displacement parameters (Å^2^)

**Table:**

	*x*	*y*	*z*	*U*_iso_*/*U*_eq_	
S1	−0.87437 (3)	0.64372 (3)	0.33879 (5)	0.02064 (13)	
N1	−0.66924 (10)	0.51441 (11)	0.17680 (17)	0.0192 (3)	
H1A	−0.6294 (19)	0.443 (2)	0.140 (3)	0.05*	
H1B	−0.6319 (19)	0.583 (2)	0.135 (3)	0.05*	
C2	−0.79042 (12)	0.51613 (11)	0.27634 (18)	0.0154 (3)	
N3	−0.84841 (10)	0.41073 (10)	0.32669 (17)	0.0177 (2)	
H3	−0.928 (2)	0.408 (2)	0.406 (3)	0.05*	
N4	−0.77834 (10)	0.30715 (10)	0.28202 (16)	0.0170 (2)	
C5	−0.84105 (12)	0.21003 (12)	0.32281 (19)	0.0168 (3)	
H5	−0.9299	0.2125	0.38	0.02*	
C6	−0.77714 (12)	0.09669 (12)	0.28214 (18)	0.0165 (3)	
C7	−0.82981 (13)	−0.01458 (12)	0.3055 (2)	0.0203 (3)	
H7	−0.9172	−0.0336	0.3521	0.024*	
N8	−0.73164 (11)	−0.09260 (11)	0.24822 (17)	0.0214 (3)	
H8	−0.743 (2)	−0.172 (2)	0.251 (3)	0.05*	
C9	−0.62411 (13)	−0.02867 (13)	0.1925 (2)	0.0218 (3)	
H9	−0.5421	−0.0627	0.1467	0.026*	
N10	−0.64591 (10)	0.08743 (10)	0.20915 (17)	0.0191 (3)	
O21	−0.50826 (10)	0.29013 (9)	−0.04044 (17)	0.0250 (2)	
H21A	−0.552 (2)	0.3077 (19)	−0.118 (3)	0.05*	
H21B	−0.552 (2)	0.237 (2)	0.038 (3)	0.05*	

### Atomic displacement parameters (Å^2^)

**Table:**

	*U*^11^	*U*^22^	*U*^33^	*U*^12^	*U*^13^	*U*^23^
S1	0.01797 (19)	0.01144 (19)	0.0285 (2)	0.00167 (11)	0.00184 (13)	0.00089 (13)
N1	0.0159 (5)	0.0133 (6)	0.0254 (6)	−0.0002 (4)	0.0004 (4)	0.0000 (5)
C2	0.0159 (6)	0.0146 (6)	0.0152 (6)	0.0002 (5)	−0.0030 (4)	−0.0003 (5)
N3	0.0154 (5)	0.0117 (6)	0.0224 (6)	0.0018 (4)	0.0025 (4)	−0.0001 (4)
N4	0.0174 (5)	0.0131 (6)	0.0185 (6)	0.0028 (4)	−0.0009 (4)	−0.0010 (4)
C5	0.0158 (6)	0.0158 (7)	0.0178 (6)	−0.0005 (5)	−0.0022 (5)	−0.0003 (5)
C6	0.0171 (6)	0.0162 (7)	0.0158 (6)	−0.0002 (5)	−0.0030 (5)	−0.0002 (5)
C7	0.0207 (6)	0.0163 (7)	0.0230 (7)	−0.0019 (5)	−0.0034 (5)	−0.0006 (5)
N8	0.0279 (6)	0.0114 (6)	0.0240 (6)	−0.0005 (5)	−0.0046 (5)	−0.0013 (5)
C9	0.0224 (7)	0.0167 (7)	0.0240 (7)	0.0032 (5)	−0.0015 (5)	−0.0012 (5)
N10	0.0177 (5)	0.0139 (6)	0.0233 (6)	0.0013 (4)	−0.0006 (4)	−0.0001 (4)
O21	0.0221 (5)	0.0221 (6)	0.0298 (6)	−0.0038 (4)	−0.0047 (4)	0.0068 (4)

### Geometric parameters (Å, º)

**Table:**

S1—C2	1.6986 (13)	C6—C7	1.3685 (18)
N1—C2	1.3309 (17)	C6—N10	1.3955 (16)
N1—H1A	0.92 (2)	C7—N8	1.3630 (18)
N1—H1B	0.88 (2)	C7—H7	0.95
C2—N3	1.3480 (17)	N8—C9	1.3453 (18)
N3—N4	1.3844 (15)	N8—H8	0.90 (2)
N3—H3	0.90 (2)	C9—N10	1.3264 (18)
N4—C5	1.2816 (17)	C9—H9	0.95
C5—C6	1.4456 (18)	O21—H21A	0.83 (2)
C5—H5	0.95	O21—H21B	0.87 (2)
			
C2—N1—H1A	119.8 (13)	C7—C6—C5	127.99 (12)
C2—N1—H1B	118.5 (14)	N10—C6—C5	122.42 (12)
H1A—N1—H1B	121.2 (19)	N8—C7—C6	106.20 (12)
N1—C2—N3	117.63 (12)	N8—C7—H7	126.9
N1—C2—S1	123.18 (10)	C6—C7—H7	126.9
N3—C2—S1	119.18 (10)	C9—N8—C7	107.62 (12)
C2—N3—N4	118.96 (11)	C9—N8—H8	129.8 (14)
C2—N3—H3	120.5 (14)	C7—N8—H8	122.6 (14)
N4—N3—H3	119.7 (14)	N10—C9—N8	112.12 (12)
C5—N4—N3	115.68 (11)	N10—C9—H9	123.9
N4—C5—C6	120.23 (12)	N8—C9—H9	123.9
N4—C5—H5	119.9	C9—N10—C6	104.47 (11)
C6—C5—H5	119.9	H21A—O21—H21B	106 (2)
C7—C6—N10	109.59 (11)		
			
N1—C2—N3—N4	3.30 (18)	C5—C6—C7—N8	−179.86 (12)
S1—C2—N3—N4	−177.56 (9)	C6—C7—N8—C9	−0.07 (15)
C2—N3—N4—C5	−175.73 (11)	C7—N8—C9—N10	−0.24 (16)
N3—N4—C5—C6	180.00 (11)	N8—C9—N10—C6	0.43 (15)
N4—C5—C6—C7	−175.90 (13)	C7—C6—N10—C9	−0.46 (15)
N4—C5—C6—N10	3.9 (2)	C5—C6—N10—C9	179.72 (12)
N10—C6—C7—N8	0.33 (15)		

### Hydrogen-bond geometry (Å, º)

**Table:**

*D*—H···*A*	*D*—H	H···*A*	*D*···*A*	*D*—H···*A*
N1—H1*A*···O21	0.92 (2)	2.34 (2)	3.2325 (16)	163.2 (18)
N8—H8···S1^i^	0.90 (2)	2.51 (2)	3.3334 (13)	153.0 (18)
O21—H21*B*···N10	0.87 (2)	2.17 (2)	3.0399 (15)	172 (2)

Symmetry code: (i) *x*, *y*−1, *z*.

## References

[bb1] Alomar, K., Landreau, A., Allain, M., Bouet, G. & Larcher, G. (2013). *J. of Inorg. Biochem.* **126**, 76–83. 10.1016/j.jinorgbio.2013.05.013 23792913

[bb2] Altomare, A., Burla, M. C., Camalli, M., Cascarano, G. L., Giacovazzo, C., Guagliardi, A., Moliterni, A. G. G., Polidori, G. & Spagna, R. (1999). *J. Appl. Cryst.* **32**, 115–119.

[bb3] Bruker (2006). *APEX2*, *SAINT* and *SADABS* Bruker AXS Inc., Madison, Wisconsin, USA.

[bb4] Farrugia, L. J. (1999). *J. Appl. Cryst.* **32**, 837–838.

[bb5] Farrugia, L. J. (2012). *J. Appl. Cryst.* **45**, 849–854.

[bb6] Finch, R. A., Liu, M., Grill, S. P., Rose, W. C., Loomis, R., Vasquez, K. M., Cheng, Y. & Sartorelli, A. C. (2000). *Biochem. Pharmacol.* **59**, 983–991. 10.1016/s0006-2952(99)00419-0 10692563

[bb7] Sheldrick, G. M. (2008). *Acta Cryst.* A**64**, 112–122. 10.1107/S0108767307043930 18156677

