# Supplementary material for: (E)-2-[(1H-Imidazol-4-yl)methyl­idene]hydrazinecarbo­thio­amide monohydrate
Source: Acta Crystallogr Sect E Struct Rep Online. 2013 Aug 23;69(Pt 9):o1469. doi: 10.1107/S1600536813022927 (PMC3884473; doi:10.1107/S1600536813022927)
Supplement: Supplementary file 3 [file e-69-o1469-Isup3.docx]

[**Figure 1**](readonly)

The molecular structure of the title compound with displacement ellipsoids drawn at the 50% probability level

[**Figure 2**](readonly)

Crystal structure of the title compound in b view along the *b* axis with hydrogen bonds shown as dashed lines
